# Flake (NH_4_)_6_Mo_7_O_24_/Polydopamine as a High Performance Anode for Lithium Ion Batteries

**DOI:** 10.3390/ma14051115

**Published:** 2021-02-27

**Authors:** Ying Xie, Xiang Xiong, Kai Han

**Affiliations:** State Key Laboratory for Powder Metallurgy, Powder Metallurgy Research Institute, College of Chemistry and Chemical Engineering, Central South University, Changsha 410083, China; 213109@csu.edu.cn

**Keywords:** (NH_4_)_6_Mo_7_O_24_, flake, lithium ion battery, anode, polydopamine

## Abstract

Ammonium molybdate tetrahydrate ((NH_4_)_6_Mo_7_O_24_) (AMT) is commonly used as the precursor to synthesize Mo-based oxides or sulfides for lithium ion batteries (LIBs). However, the electrochemical lithium storage ability of AMT itself is unclear so far. In the present work, AMT is directly examined as a promising anode material for Li-ion batteries with good capacity and cycling stability. To further improve the electrochemical performance of AMT, AMT/polydopamine (PDA) composite was simply synthesized via recrystallization and freeze drying methods. Unlike with block shape for AMT, the as-prepared AMT/PDA composite shows flake morphology. The initial discharge capacity of AMT/PDA is reached up to 1471 mAh g^−1^. It delivers a reversible discharge capacity of 702 mAh g^−1^ at a current density of 300 mA g^−1^, and a stable reversible capacity of 383.6 mA h g^−1^ is retained at a current density of 0.5 A g^−1^ after 400 cycles. Moreover, the lithium storage mechanism is fully investigated. The results of this work could potentially expand the application of AMT and Mo-based anode for LIBs.

## 1. Introduction

In recent years, lithium ion batteries (LIBs) have been recognized as the solution to many challenges due to the booming development of electric vehicles and energy storage industries [1,2,3,4]. Their benefits include higher specific energy and a longer lifespan, and they are also more cost-effective. Anode materials with lower work potential and higher energy/power density have been widely studied to improve the integral performance of LIBs [5]. Three main types of anode materials, including carbon-based material (such as graphite [6], carbon nanotubes [7], and graphene [8]), transition metal oxides (such as Fe_2_O_3_ [9] and Co_3_O_4_ [10]), and elementary substances (Si [11], Sn [12]) have drawn extensive attention, and great progress has been made in recent year to promote their practical application. However, to meet the high energy storage requirements of electric vehicles and smart devices, anode materials with advantages of high specific capacity, long-term cycling stability, and facile synthesis approach that can be easily scaled up are still urgently needed.

Polyoxometalate is a type of compound obtained via the condensing and recrystallizing process of the oxoacid ion of a pro-transition metal with a d^0^ electronic configuration in a solution. Ammonium molybdate tetrahydrate (AMT), one of the polyoxometalates with the advantage of industrial low cost, has been studied extensively [13]. Researchers have mainly used AMT to synthesize MoS_2_ or MoO*_x_* (x = 2, 3) via a series of complicate procedures [14]. For example, Luo et al. [15] synthesized MoO_2_@carbon nanofiber using AMT as a precursor. When applied as an anode for LIBs, MoO_2_@carbon nanofiber composite showed a discharge capacity of 762.7 mAh g^−1^ after 100 cycles at a current density of 50 mA g^−1^. Chang et al. [16] prepared MoS_2_-graphene composite via a solution-phase approach. MoS_2_ in the composites possessed a typical layered structure with a sheet size of 100–200 nm. The composite exhibited a specific capacity of about 900 mAh g^−1^ at a current density of 1000 mA g^−1^. Furthermore, hexagonal Mo based cluster compounds such as Mn_2_Mo_3_O_8_ and Fe_2_Mo_3_O_8_ have also been developed for LIB anodes in recent years [17,18,19]. Although the above reports achieve high capacity Mo-based oxide or sulfides, the synthesis procedures of these nanostructured materials are generally complex and need to be precisely controlled. Inspired by the conversion mechanism of Mo-based compounds during the lithiation and delithiation process in LIBs, our concern is, can AMT be directly used as an anode material for LIBs? If so, the complex synthesis process from AMT to Mo-oxides/sulfides would be unnecessary. However, the electrochemical lithium storage ability of AMT itself is still unclear so far. Herein, we initially investigate the lithium storage properties of AMT and focus on its electrochemical performance improvement. 

In addition, polydopamine (PDA) is widely employed as a coating material owing to its natural non-toxicity and super adhesion [20]. The multiple N types derived from the amine functional group of PDA could improve the electronic conductivity and help Li^+^ transportation [21]. Interestingly, it has been found that pure PDA can show some lithium storage ability, and the electrochemical lithium storage ability of PDA could be enhanced by molecule adjustments such as oxidation and heat treatment [22,23]. Therefore, PDA was applied to modify the surface of AMT in this work. The flake AMT/PDA sheets were synthesized by a low-temperature treatment process. Differing from the large block morphology of the pristine AMT, AMT/PDA shows a flake structure. The pure AMT is confirmed to store lithium reversibly under electrochemical conditions. The electrochemical performance of AMT could be significantly enhanced by synthesis of AMT/PDA. The initial discharge capacity of AMT/PDA is reached up to 1471 mAh g^−1^, and the capacity remains 702 mAh g^−1^ at a current density of 300 mA g^−1^ and 383.6 mA h g^−1^ at a current density of 0.5 A g^−1^ after 400 cycles. The results can potentially expand the application of AMT and Mo-based anodes for LIBs. 

## 2. Materials and Methods

### 2.1. Material Synthesis 

In a typical experiment, 20.0 mg of dopamine was weighted and transferred into a conical flask. Afterwards, 1.0 g AMT was also added to the above conical flask, and 50 mL of deionized water was poured in. Subsequently, the pH of the solution was adjusted to 8.5 by adding Tris-HCl buffer solution. The solution was stirred vigorously at 40 °C for 24 h. Finally, the obtained composites were freeze dried to gain the resultant product AMT/PDA. 

### 2.2. Material Characterizations

The crystalline structure of all the samples were detected by X-ray diffraction (XRD) technology with a Bruker D8 advance (Bruker, Billerica, MA, USA) (Cu Kα, λ = 1.5418 Å). The chemical environment of various elements in the samples was measured via X-ray photoelectron spectroscopy (XPS) with a Thermo Scientific ESCALAB 250Xi (ThermoFisher Scientific, Waltham, MA, USA). The morphology of the AMT freeze dried AMT and AMT/PDA was observed by employing scan electron microscopy (SEM, Nova Nano230, FEI Company, Hillsboro, OR, USA). The chemical information of the AMT and AMT/PDA was collected by a Fourier transform infrared (FTIR) spectrometer (FTIR-650, Gangdong Instrument Company, Tianjin, China). The weight loss of the sample was measured by thermogravimetric analysis (TGA) (STA2500, Netzsch, Waldkraiburg, Germany) under an Ar atmosphere from room temperature to 500 °C with a heating rate of 5 °C/min.

### 2.3. Electrochemical Measurements

The electrochemical properties of AMT, freeze dried AMT, and AMT/PDA electrodes were investigated by assembling a CR2032 coin cell (MIT Company, Shenzhen, China). The active material AMT or freeze dried AMT, AMT/PDA, or PDA, polyvinylidenefluoride(PVDF), and carbon black super-P was vigorously stirred at a mass ratio of 7:2:1 for 12 h and cast on copper foil to obtain the working electrodes, which were dried in an oven at 60 °C for 12 h. The CR2032 coin cells were assembled in an argon-filled glove box with lithium metal as the counter electrode, with H_2_O and O content below 0.1 ppm. The ingredient of electrolyte is comprised of 1.0 M LiPF_6_ solution containing ethylene carbonate/dimethyl carbonate (EC/DEC, 1:1 in volume). Glass-fiber (Whatman, CF-F, Maidstone, UK) was applied as the separator. The electrochemical performance for all the electrodes was evaluated by galvanostatic charge/discharge test (LAND CT2000, Wuhan LAND electronics Co., Wuhan, China) in the voltage range from 0.005 V to 2.5 V vs. Li^+^/Li. The area mass loading for every electrode was about 1.2 mg cm^−2^. Cyclic voltammetry (CV) curves for AMT and AMT/PDA were collected on a CHI660E electrochemical workstation between 0.005 V and 2.5 V at a scan rate of 0.1 mV s^−1^. Electrochemical impendence spectra (EIS) of the AMT and AMT/PDA electrodes before and after cycles were measured with an electrochemical workstation (CHI660E) (Chenhua Instrument Company, Shanghai, China) between 100 kHz and 0.01 Hz. 

## 3. Results and Discussion

**Figure 1:**
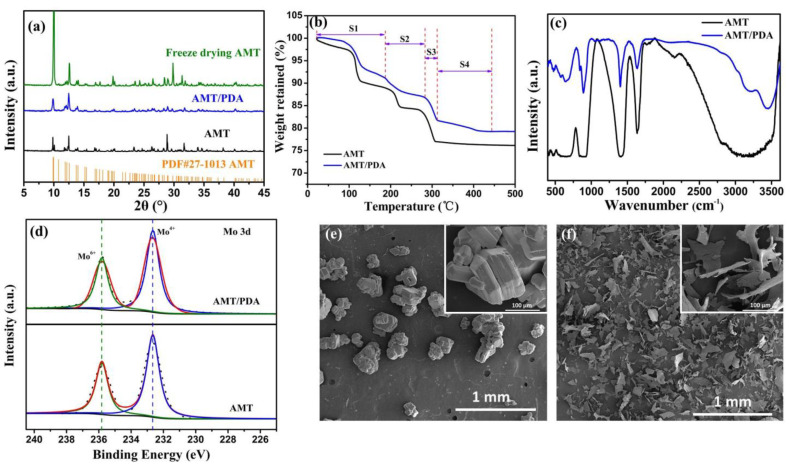
(**a**) XRD patterns of ammonium molybdate tetrahydrate (AMT), AMT/polydopamine (AMT/PDA), and AMT-Cryodesiccation, (**b**) TGA curves of AMT and AMT/PDA, (**c**) FTIR spectra of AMT and AMT/PDA, (**d**) High-resolution XPS spectrum of Mo 3d of AMT and AMT/PDA. SEM images of (**e**) AMT, (**f**) AMT/PDA.

The XRD patterns of the AMT, freeze dried AMT, and AMT/PDA samples, as well as the standard card of the AMT, are shown in Figure 1a. Because PDA is an amorphous structure, AMT/PDA presents no obvious new peaks compared with AMT and freeze dried AMT [24]. This also results in much weaker characteristic diffraction peaks for AMT/PDA than for AMT and freeze dried AMT. Furthermore, the peak intensity of freeze dried AMT is much higher than that of AMT. This is probably due to the presence of crystal water in AMT, which could be removed after the freeze drying process. Meanwhile, Figure 1b shows the thermogravimetric analysis of the AMT and AMT/PDA. Both AMT and AMT/PDA samples underwent three weight loss phases when the temperature was ≤300 °C. The lost weight resulted from H_2_O in step one (S1), H_2_O and NH_3_ in step two (S2), and NH_3_ in step three (S3). Corresponding lost content are 8.82%, 4.31%, and 5.13%, respectively, which is consistent with the calculation of the integrant weight of N, H, and O in AMT. There was another weight loss (S4) when the temperature increased to about 410 °C. This could be as a result of the pyrolysis of PDA, whose corresponding content is 2.5%, which further illustrates the existence of the PDA [25].

To verify the chemical information of AMT/PDA composite, Fourier transform infrared (FTIR) characterization was carried out. The bending vibration band of the N-H functional group for AMT and AMT/PDA samples locates at about 1639 cm^−1^. Meanwhile, the peak at about 1397 cm^−1^ for the AMT/PDA sample is narrower and sharper compared to that of AMT, which could be assigned to the shear vibration superposition band of C = C in the benzene ring and the N-H functional group. Moreover, there are two broad peaks at about 3400 and 3200 cm^−1^. This could be considered as the symmetric and asymmetric stretching vibration absorption of the N-H functional group. The above results confirm the presence of PDA in the AMT/PDA composite [24].

The XPS analysis was used to obtain the chemical environment of several elements (such as Mo and O). Four main strong expected XPS peaks, Mo 3d at 233.3 eV, Mo 3p_3/2_ at 399.3 eV, Mo 3p_1/2_ at 417.2 eV, and O 1s at 530.9 eV, are shown in Figure S1a. Figure 1d shows the fitting pattern of the Mo 3d XPS spectra, which can give more detailed valence state of Mo cations in the AMT/PDA and AMT samples. There are two peaks located at 232.7 eV and 235.8 eV, which could be matched with the tetravalent (Mo^4+^) and hexavalent (Mo^6+^) states [26]. There is no significant difference in binding energy between AMT and AMT/PDA. This shows that the chemical environment of the Mo cations has not been changed via low-temperate treatment. The above results are consistent with those of XRD. The fitting C 1s and O 1s XPS spectra of AMT and AMT/PDA are shown in Figure S1b,c. There is a clear XPS peak at 530.5 eV as for the AMT and AMT/PDA samples, which belongs to the chemical environment of oxygen in MoO*_x_* (2 < *x* < 3) [27]. It is intriguing to note for the AMT/PDA samples that there is an XPS peak at 532.0 eV, which is assigned to the C-O bond [8]. The existence of the C-O bond revealed that PDA exists in the AMT. Moreover, the Mo atomic percentage for AMT/PDA (35.32%) is less than that of AMT (41.63%) (Table S1), which could be ascribed to the coverage role of PDA.

It has been reported that the freeze drying process might effectively rebuild the morphology structure of the material [28]. SEM was employed to obtain the morphology of the AMT and AMT/PDA samples, and the results are shown in Figure 1e,f. The shape of AMT has a cuboid-like and rock-like appearance, with a slick surface (Insets in Figure 1e,f). The size of the cuboid-like AMT is approximately ~200 μm. The morphology of the freeze dried AMT is still cuboid-like, with about 5 μm thickness (Figure S2a). There are significant changes for the AMT/PDA regarding morphology, transforming from rock-like shapes to sheets (Figure 1f). The length, width, and thickness of AMT/PDA is, on average, about 100, 60, and 1.2 μm, respectively, according to Figure S2b–d.

**Figure 2:**
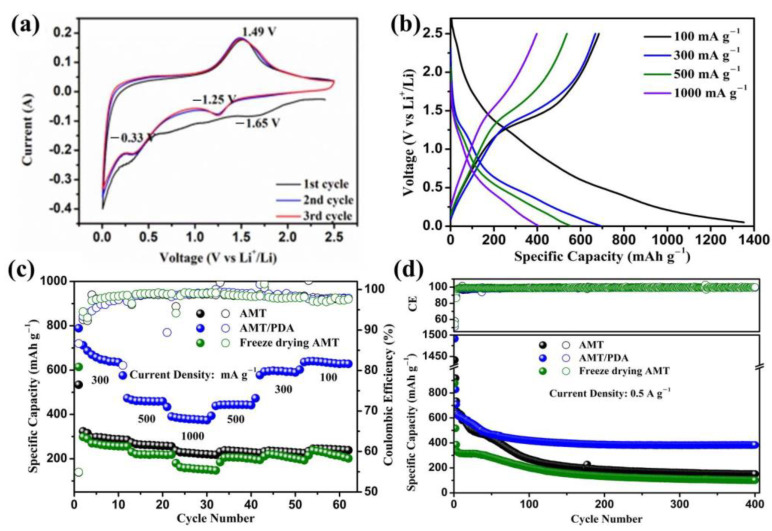
(**a**) Cyclic voltammetry curves of the AMT/PDA electrode from 0.005V to 2.5 at a scanning rate of 0.1 mV s^−1^, (**b**) Charge-discharge voltage profiles of the AMT/PDA electrode, (**c**) Rate capability, (**d**) cycling performances of the AMT, AMT/PDA, and freeze dried AMT electrode

Sheet morphology, as well as the presence of PDA, may lead to differences in electrochemical performances. The corresponding electrochemical performances for all the samples have been investigated and are shown in Figure 2.

The delicate charge/discharge process was measured with cyclic voltammetry at a scanning rate of 0.1 mV s^−1^ from 0.005~2.5 V vs. Li^+^/Li, and the results are shown in Figure 2a and Figure S3. AMT, freeze dried AMT, and AMT/PDA exhibit very similar sharpness in the CV curves, suggesting similar electrochemical lithium storage properties, especially reversible oxidation and reduction behavior. This also demonstrates that the main electrochemical lithium storage ability contribution for AMT/PDA comes from AMT. During the first discharge process, a broad cathodic peak appearing at around 1.65 V illustrates the formation of the solid electrolyte interphase (SEI) film, and it disappears in the subsequent cycles [29]. The Mo^6+^ state converts to the Mo^4+^ state when the potential further decreases to 1.25 V. Finally, the Mo^4+^ reacts with Li^+^ to form certain stable composites. Meanwhile, a stable anodic peak at about 1.49 V is observed. It is assigned to the reversible conversion reaction of the Li/Mo composites back to oxides (MoO_3_/MoO_2_) [30,31]. Figure 2b shows the charge/discharge voltage profiles at different current densities for the AMT/PDA electrode, with a charge platform range from 1.25 to 1.75 V. Figure 2c,d shows the rate capability and cycling performances of the AMT, freeze dried AMT, and AMT/PDA electrodes, respectively. The AMT/PDA electrode exhibited obvious rate capability improvement compared to the AMT and the freeze dried AMT electrodes from Figure 2c. At a current density of 300 mA g^−1^, AMT/PDA delivered a reversible discharge capacity of 702 mAh g^−1^, which was only about 300 mAh g^−1^ for AMT and freeze dried AMT. With a current density increase to 500 and 1000 mA g^−1^, capacities of 465 mAh g^−1^ and 397 mAh g^−1^, respectively, were maintained. After the current density went back to 500 and 300 mA g^−1^, the capacity gradually recovered, suggesting the good reversibility of the AMT/PDA electrode. The AMT, freeze dried AMT, and AMT/PDA electrodes delivered initial discharge capacities of 1331.8, 878.6, and 1471 mAh g^−1^, respectively, at a current density of 100 mA g^−1^ with an initial coulombic efficiency (ICE) of 55.42%, 54.28%, and 51.31%, respectively. As shown in Figure S4, the pure PDA electrode showed an irreversible discharge capacity of about 170 mAh g^−1^ in the first cycle. This is the reason for the lower ICE of AMT/PDA compared to the AMT and freeze dried AMT electrodes. However, in the subsequent cycles, the pure PDA electrode only delivered a reversible stable capacity of less than 20 mAh g^−1^, suggesting that the most capacity contribution in AMT/PDA comes from AMT. The specific capacity of the AMT/PDA electrode maintained 383.3 mA h g^−1^ after 400 cycles at a current density of 0.5 A g^−1^, which was the best among the prepared electrodes (Figure 2d). Meanwhile, a long-term galvanostatic test at an even higher current density of 1 A g^−1^ was carried out to compare the stability of the materials under high current density conditions (Figure S5). The AMT/PDA electrode exhibited the most stable performance (267.7 mAh g^−1^ after 1000 cycles). There is obvious capacity decay for all electrodes. However, there is a difference of attenuation cycles (50 cycles for the AMT/PDA electrode, 100 cycles for the AMT electrode). The decay could be related to the structure of the AMT. MoO_3_ might collapse and pulverize during the cycle phase [30]. When the surface of the AMT is coated by PDA, the PDA can be regarded as a protective layer to alleviate the structure collapse. The charge/discharge voltage profiles of AMT and AMT/PDA during long-term cycling shown in Figure S6 further confirm the similar lithium storage mechanism from the similar voltage plateau. The voltage profiles also indicate the higher cycling stability of AMT/PDA compared AMT. As a result, the AMT/PDA composite exhibits much higher capacity than that of the commercialized graphite anode, particularly at current densities over 0.5 A g^−1^ [32]. In addition, the rate capability and cycling stability of AMT/PDA is much better than the pure Mo-based oxides [15]. Therefore, considering the facile synthesis procedure and the micro-size scale, the AMT/PDA could be regarded as a promising anode material for high energy and high power lithium ion batteries. However, similar to the other high theoretical capacity anode materials, including silicon and transition metal oxides/sulfides [33], the ICE of AMT/PDA is still to be improved for future practical application. 

**Figure 3:**
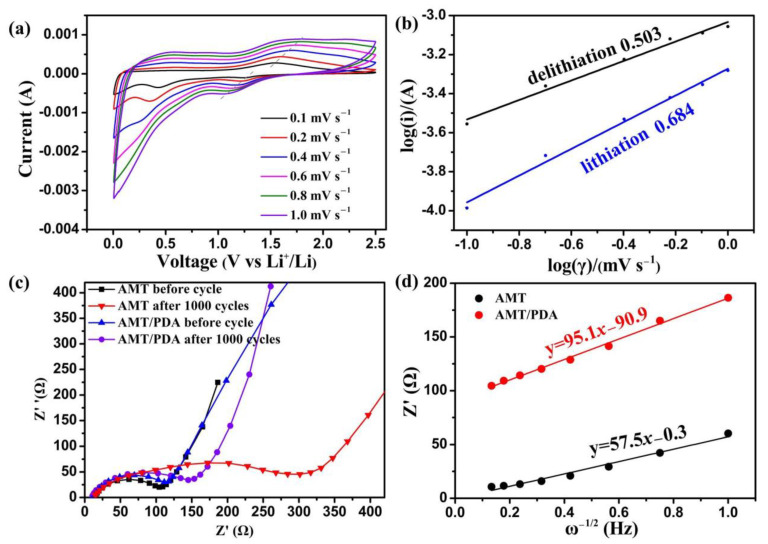
(**a**) Cyclic voltammograms of AMT/PDA electrode at different sweep rate from 0.005 V to2.5 V, (**b**) Plot of log(i) vs. log(v) from the cathodic segment at different scanning rates from Figure 3a, (**c**) EIS spectra of the fresh and cycled AMT and AMT/PDA electrodes, (**d**) Plot of Z’ vs. ω^−1/2^, derived from Figure 3c.

In order to understand the electrochemical characterization of AMT/PDA electrodes for lithium ion storage, the anodic/cathodic peak current at different scanning sweeps was selected. The results are shown in Figure 3a.

The mathematical relationship between the response peak current and the scanning rate could be descripted as the following (Equation (1)):

I = aν^b^ (log(i) = blog(ν) + log(a)) (1)

where i is the current, ν represents the scanning rate, and a and b are adjustable parameters. The working mechanism of the electrode is controlled by the value of b, which is attributed to the fact that i and ν are known with regard to an explicit scanning process. When the value of b is closer to 1, this indicates that the capacitance property of the electrode material is superior. On the contrary, the closer the value of b is to 0.5, the better the battery property of the electrode material [34,35] The value of b, the slope of the linear line in Figure 3b, is equal to 0.684 and 0.503, respectively, for the lithiation/delithiation process. The b value is close to 0.5, indicating that the working mechanism of the AMT/PDA electrode does not belong to the capacitance property.

According to the literature reports and the analysis of CV curves, the position of cathodic/anodic peaks and the shape of the voltage profiles are similar to the initial lithiation of the oxide-based anode material of molybdenum in LIBs [30,31,36]. So a possible reaction equation (Equations (2)–(5)) is proposed and shown as following:

AMT + (20 − 2*x*)Li^+^ + (14 − 2*x*)e^−^ →6NH^4+^ + *x*MoO_3_ + (7 − x)MoO_2_ + (6 − *x*)Li_2_O + 8LiOH (2)

MoO_3_ + *y*Li^+^ + *y*e^−^ →Li*_y_*MoO_3_ (3)

Li*_y_*MoO_3_ + zLi^+^ + ze^−^ →Mo^0^ + 3Li_2_ (4)

Mo^0^ + 3Li_2_O →MoO_3_ + 6e^−^ + 6Li (5)

Equation (2) presents an irreversible conversion reaction, and the others are highly reversible for lithium storage. This could be used to explain the low ICE of every electrode. Electrochemical impedance spectroscopy (EIS) was also employed to explain the excellent electrochemical performance of AMT/PDA. The corresponding Nyquist plots are shown in Figure 3c. The diameter of a semicircle could be used to describe the electrochemical reaction resistance of the electrode [37]. The electrochemical reaction resistance of the cycled AMT/PDA electrode (141.3 Ω) is much lower than that of the cycled AMT electrode (314.6 Ω), demonstrating the successful modifying role. As shown in Figure 3d, the linear relationships between Z’ and ω^−1/2^ in the low frequency are fitting. The σ represented by the slope of a line is calculated by Equation (S1) (seen in Supplementary Data). According to Equation (S2) (seen in Supplementary Data), a minor σ value indicates faster ion diffusion [38,39,40]. The slope of the cycled AMT/PDA electrodes is lower than in the AMT electrodes, which suggests better Li-ion diffusion in the internal AMT/PDA electrodes. The results further confirm that AMT/PDA possesses the faster charge transfer and Li-ion diffusion kinetics, leading to excellent electrochemical performance.

**Figure 4:**
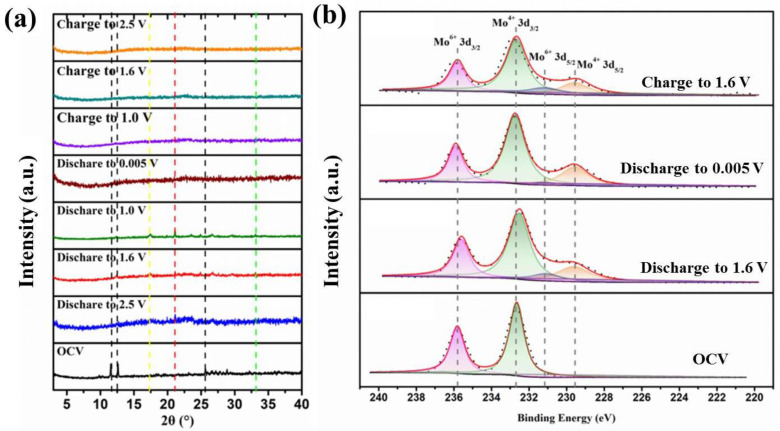
(**a**) E*x*-situ XRD patterns of the AMT/PDA electrode with different charge-discharge state, (**b**) E*x*-situ XPS spectrums of Mo 3d for the AMT/PDA electrode with different charge-discharge state

In order to further comprehend the behaviors of the lithiation and delithiation processes for the AMT/PDA electrode, ex-situ XRD and XPS measurement were conducted, as shown in Figure 4. For the new assembled battery, two relatively strong diffraction peaks, located at 12.4° and 25.5°and several weak diffraction peaks between 25.5°and 30° were discovered (Figure 4a), and these are in agreement with the standard card of AMT. The diffraction peaks at 12.4° and 25.6° disappear, and other peaks come out when discharged to 1. 6-1. 0 V. The new diffraction peaks at 17.5°, 21.1°, 23.5°, 24.9°, 26.7°, and 33.2° could be consistent with the standard card of lithium molybdenum oxide (Li_4_Mo_5_O_17_) (PDF#25-0492) and molybdenum trioxide (PDF#05-0506). There is no diffraction peak during the latter lithiation and delithiation process, indicating that the crystal structure of the electrode material may be converted to an amorphous state. As shown in Figure 4b, there are two main valence state forms of Mo^6+^ (3d_3/2_, 235.8 eV) and Mo^4+^ (3d_3/2_, 232.7 eV) for Mo. When the electrode is discharged to 1.6V and 0.005 V, the relative content of Mo^4+^ (3d_5/2_, 229.6 eV) increases. However, the relative content of Mo^6+^ (3d_5/2_, 231.2 eV) increases first and then decreases [30]. The above results indicate the redox of the Mo element, changing from Mo^6+^ to Mo^4+^. When the electrode was charged to 1.6 V again, the Mo (3d_5/2_, 231.2 eV) appears, showing excellent reversible transformation.

## 4. Conclusions

In summary, the electrochemical lithium storage performance of AMT was first proven. To further improve its electrochemical performance, the flake AMT/PDA composite was successfully synthesized via recrystallization and freeze drying methods. The working mechanism of AMT/PDA as an anode material is its battery behavior. The AMT/PDA delivers a high initial discharge capacity of 1471 mAh g^−1^, and a capacity of 702 mAh g^−1^ at a current density of 300 mA g^−1^ is retained. During long-term cycling, stable capacities of 383.6 mA h g^−1^ at a current density of 0.5 A g^−1^ after 400 cycles and 267.7 mAh g^−1^ at a high current density of 1 A g^−1^ after 1000 cycles were achieved, which are much higher than those of AMT and freeze dried AMT. The electrochemical performance of AMT or AMT/PDA would be further enhanced by some rational approaches such as a conducting material coat, unique structure design, and so on. The results prove that AMT can not only be used as a precursor to the synthesis of Mo-based anode materials, but itself can also reversibly store lithium without a complicated synthesis process.

## Data Availability

The article and Supplementary Materials include all data.

## Supplementary Materials

The following are available online at https://www.mdpi.com/1996-1944/14/5/1115/s1, Table S1: The atomic percentage of C, O, Mo, N elements for AMT and AMT/PDA. Figure S1: (a) XPS survey spectrum of AMT and AMT/PDA, (b) High-resolution XPS spectrum of Mo 3d of AMT and AMT/PDA. Figure S2: Cross-sectional SEM images of (a) Freeze dried AMT, (b) AMT/PDA. Statistical data of length (c) and width (d) for AMT/PDA flake. Figure S3: Cyclic voltammetry curves of (a) AMT and (b) freeze dried AMT electrodes. Figure S4: (a) Rate capability and (b) cycling performances of the PDA electrode. Figure S5: Long-term cycling performances of AMT, freeze dried AMT, and AMT/PDA electrodes. **Figure S6**: Charge/discharge voltage profiles of (a) AMT and (b) AMT/PDA during long-term cycling.
