# Supplementary material for: Flake (NH4)6Mo7O24/Polydopamine as a High Performance Anode for Lithium Ion Batteries
Source: Materials (Basel). 2021 Feb 27;14(5):1115. doi: 10.3390/ma14051115 (PMC7957530; doi:10.3390/ma14051115)
Supplement: Supplementary file 1 [file materials-14-01115-s001.pdf]

Supplementary Information

Flake $(NH_4)_6Mo_7O_{24}$/ Polydopamine as a High Performance Anode for Lithium Ion Batteries

Ying Xie, Xiang Xiong* and Kai Han*

State Key Laboratory for Powder Metallurgy, Powder Metallurgy Research Institute, College of Chemistry and Chemical Engineering, Central South University, Changsha 410083, China; 213109@csu.edu.cn

* Correspondence: xiongx@csu.edu.cn (X.X.); kaihan@csu.edu.cn (K.H.)

Calculation of lithium diffusion coefficient

Warburg diffusion coefficient (D_w) is calculated via the following equation:

$$Z' = R_\Omega + R_{ct} + \sigma\omega^{-1/2} \quad \text{(S1)}$$

$$D_w = R^2T^2/2n^4A^2F^4C^2\sigma^2 \quad \text{(S2)}$$

R is the gas constant, T is the absolute temperature, A is the surface area of the electrode, n is electron transfer number, F is Faraday constant, C is the electrolyte concentration, σ is Warburg coefficient. R_Ω is the ohmic resistance, R_{ct} is the resitance of charge transfer, and ω presents the frequency.[1-3]

Table S1 The atomic percentage of C, O, Mo, N elements for the AMT and AMT/PDA.

	**AMT**	**AMT/PDA**
**C 1s**	14.96	24.89
**O 1s**	41.63	35.32
**Mo 3d**	21.15	17.45
**N 1s**	22.25	22.34

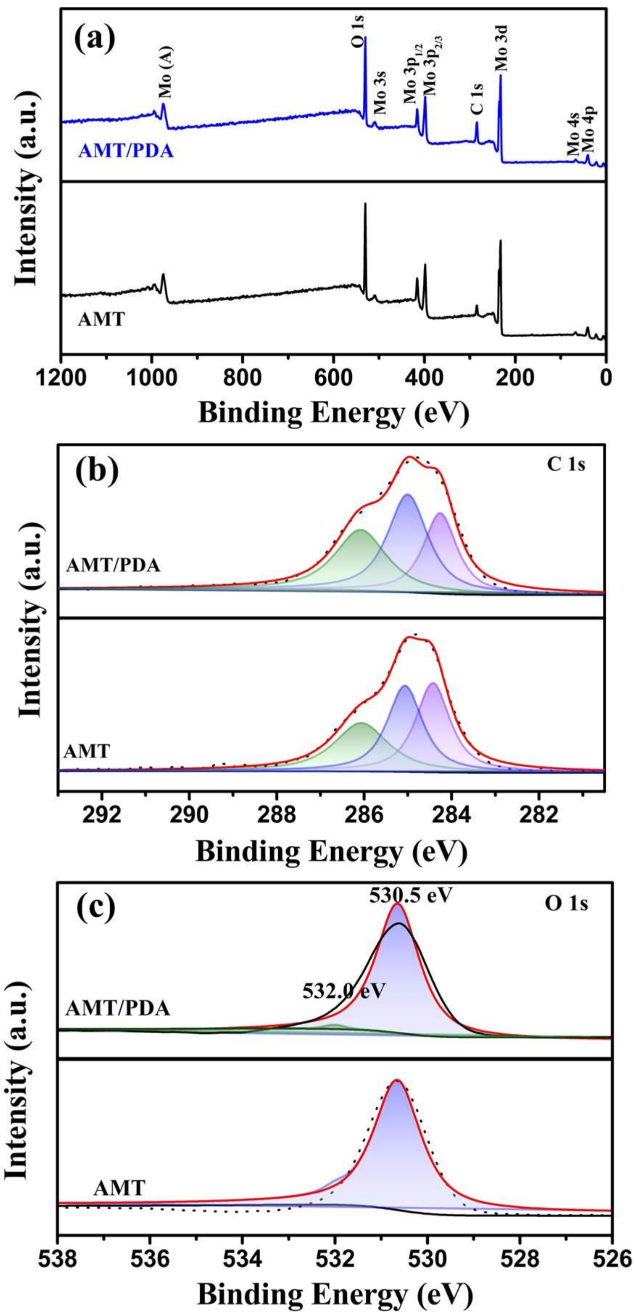

Figure S1. (a) XPS survey spectrum of AMT and AMT/PDA, High-resolution XPS spectrum of (b) C 1s and (c) O 1s of AMT and AMT/PDA.

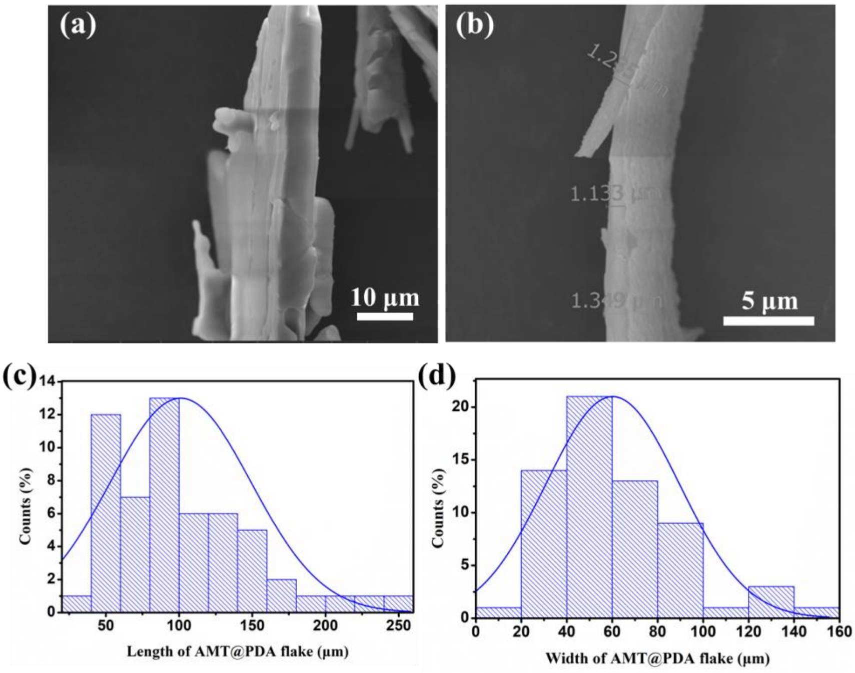

Figure S2. Cross-sectional SEM images of (a) Freeze drying AMT, (b) AMT/PDA. Statistical data of length (c) width (d) for AMT/PDA flake.

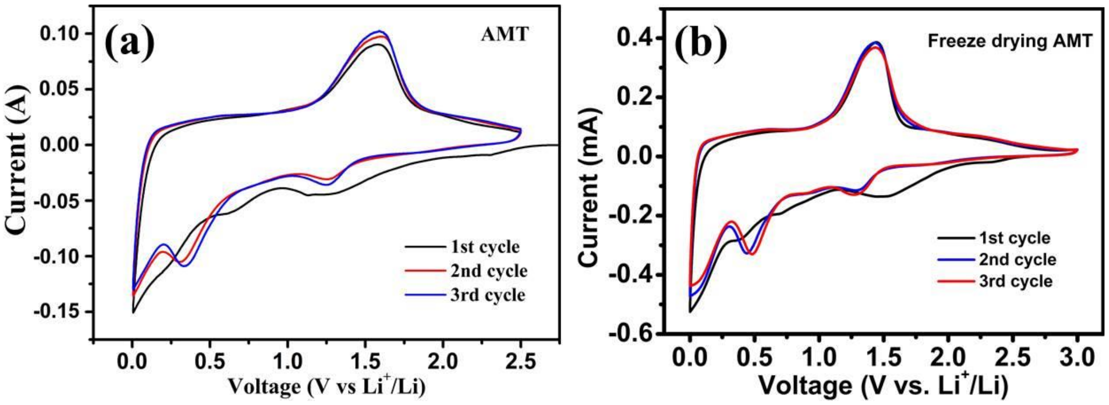

Figure S3. Cyclic voltammetry curves of (a) AMT and (b) freeze drying AMT electrodes.

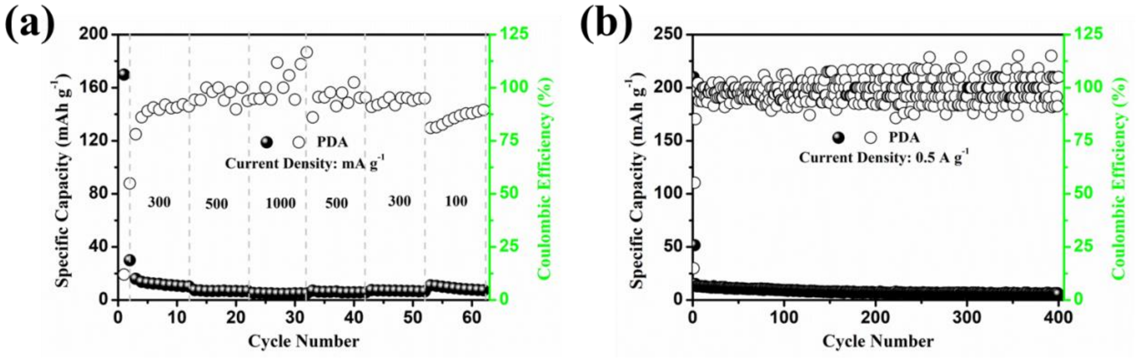

Figure S4. (a) Rate capability and (b) cycling performances of the PDA electrode.

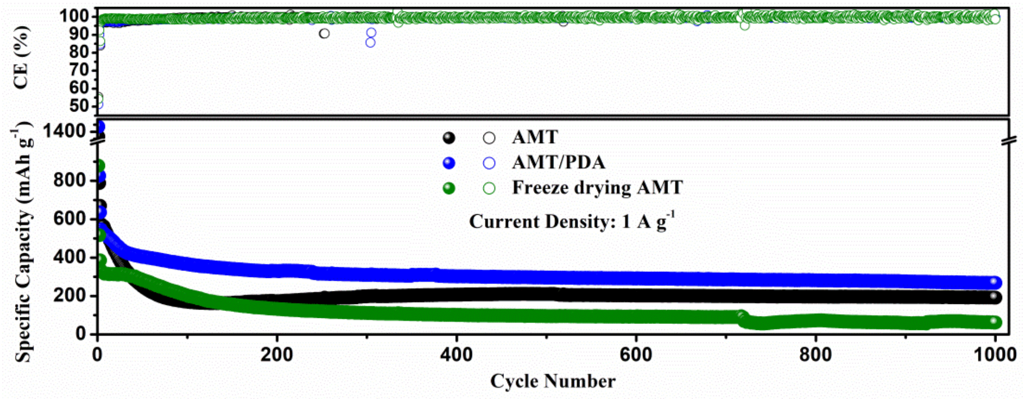

Figure S5. Long-term cycling performances of AMT, freeze drying AMT and AMT/PDA electrodes.

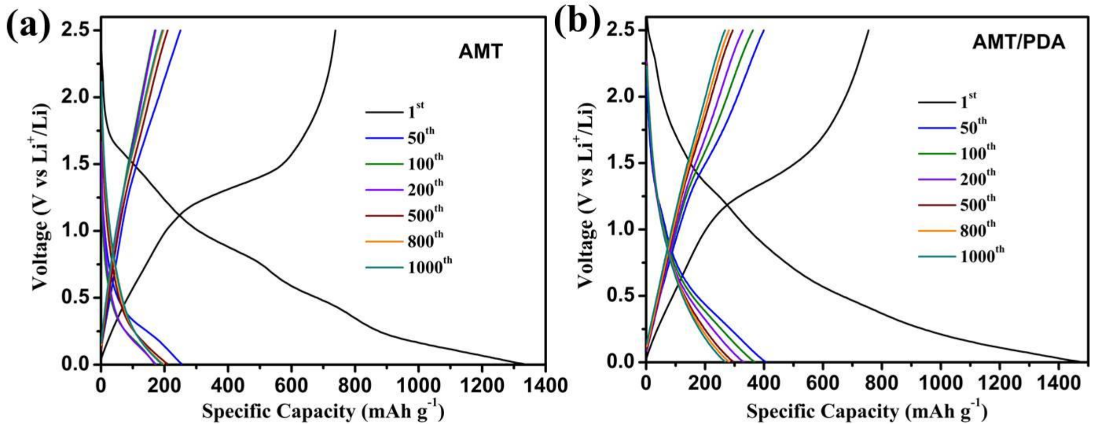

Figure S6. Charge/discharge voltage profiles of (a) AMT and (b) AMT/PDA during long-term cycling.

References

1. T. R. Chen, T. Sheng, Z. G. Wu, J. T. Li, E. H. Wang, C. J. Wu, H. T. Li,; X. D. Guo, B. H. Zhong, L. Huang, S. G. Sun, Cu^{2+} Dual-Doped Layer-Tunnel Hybrid $Na_{0.6}Mn_{1-x}Cu_xO_2$ as a Cathode of Sodium-Ion Battery with Enhanced Structure Stability, Electrochemical Property, and Air Stability. *ACS Appl. Mater. Inter.* 2018, **10**, 10147-10156.
2. H. Zhang, P. Zong, M. Chen, In Situ Synthesis of Multilayer Carbon Matrix Decorated with Copper Particles: Enhancing the Performance of Si as Anode for Li-Ion Batteries. *ACS Nano*, 2019, **13** 3054-3062.
3. Y. Fu, Q. Wei, G. Zhang, X. Wang, J. Zhang, Y. Hu, D. Wang, L. N. Zui, T. Zhou, Y. Wu, S. Sun, High-Performance Reversible Aqueous Zn-Ion Battery Based on Porous MnOx Nanorods Coated by MOF-Derived N-Doped Carbon. Adv. Energy Mater., 2018, 8, 1801445.
